# Frequency and impact of repeat colonoscopy as a treat‐to‐target approach in pediatric inflammatory bowel disease

**DOI:** 10.1002/jpr3.70110

**Published:** 2025-11-11

**Authors:** Kari Noel VanEvery, Grace Dukes, Brendan Boyle, Jennifer L. Dotson, Ross M. Maltz, Hilary K. Michel

**Affiliations:** ^1^ Division of Pediatric Gastroenterology Hepatology, and Nutrition, Nationwide Children's Hospital Columbus Ohio USA; ^2^ Department of Pediatrics The Ohio State University Wexner Medical Center Colubus Ohio USA; ^3^ Division of Pediatric Gastroenterology and Hepatology Arkansas Children's Hospital Little Rock Arkansas USA; ^4^ Department of Pediatrics University of Arkansas for Medical Sciences Little Rock Arkansas USA

**Keywords:** endoscopic healing, mucosal healing, quality improvement, therapy adjustment

## Abstract

**Objectives:**

Endoscopic healing (EH) is a treatment target in inflammatory bowel disease (IBD). However, adherence to repeat colonoscopy (RC) for EH assessment in pediatrics remains variable. We aimed to evaluate the frequency and timing of RC after diagnosis and its impact on treatment decisions at a large pediatric IBD center.

**Methods:**

We conducted a retrospective cohort study of pediatric patients diagnosed with IBD January 2019 to December 2021 using an internal patient database and electronic medical records. Data included demographics, treatments, timing of RC, reasons for no RC (if not completed), and therapy modifications following RC (medication escalations and de‐escalations). The study took place within the context of an institutional quality improvement project aimed at improving RC rates within 15 months of diagnosis.

**Results:**

Of 325 patients diagnosed via baseline colonoscopy during the study interval, 91% underwent RC; 8% within 6 months due to inadequate primary therapy response, 67% between 7 and 15 months, and 16% beyond 15 months. Among the 272 patients with a RC > 6 months postdiagnosis, therapy modification occurred in 39% (107/272), with 81% (87/107) requiring medication escalation, 16% (17/107) medication de‐escalation, and 3% (3/107) surgical intervention. Only 3% of patients declined a RC due to patient/family preference.

**Conclusions:**

Adherence to the treat‐to‐target strategy recommending RC within 15 months of diagnosis was high, exceeding previously reported pediatric rates. Only 3% of patients/families declined RC despite this being a perceived barrier toward assessment of EH. Therapy adjustments post‐RC were frequent, underscoring the importance of mucosal assessment in guiding treatment optimization.

## Abstract (highlights)

**What is Known:**

- Endoscopic healing is a key treatment target in pediatric inflammatory bowel disease (IBD).
- Repeat colonoscopy (RC) is recommended to assess endoscopic healing but adherence is variable.
- Barriers to RC include patient reluctance and provider practice variation.

**What is New:**

- Over 90% of patients at a large pediatric center completed a RC within 2 years of diagnosis.
- Nearly 40% of patients had treatment changes following colonoscopy, most commonly therapy escalation but also medication de‐escalation in select patients.
- Patient or family refusal to complete RC was very low, with only 3% declining endoscopic reassessment.
- Standardized care pathways may enhance adherence to treat‐to‐target strategies in pediatric IBD.

## 1 INTRODUCTION

The management of pediatric inflammatory bowel disease (IBD), including Crohn's disease (CD), ulcerative colitis (UC), and IBD‐unclassified (IBD‐U), prioritizes endoscopic healing (EH) as a long‐term target over symptom resolution alone. Achieving EH is associated with sustained remission, reduced complications, and improved long‐term outcomes.^1^, ^2^, ^3^, ^4^, ^5^, ^6^ Given the central role of EH in guiding management decisions, repeat colonoscopy (RC) is a critical tool for evaluating treatment response as emphasized in the STRIDE II guidelines.^2^ However, the use of RC for EH assessment remains variable in pediatric IBD populations.^7^ Reported adherence rates to RC are inconsistently characterized, with estimates ranging from 40% in adults with IBD ^8^ to nearly 90% in postresection evaluations in pediatrics.^9^ This variability may reflect both patient‐level factors and a lack of consensus on the optimal timing for repeat endoscopic assessment.^2^ It is often perceived that children and parents may view RC as burdensome, thus contributing to reluctance.^10^, ^11^ At the same time, physicians differ in when they use endoscopy to monitor disease activity, with some favoring less invasive markers, such as blood or stool testing, or possibly repeat imaging.^7^, ^12^ RC appears more readily accepted when embedded within standardized care pathways.^12^ However, few studies have evaluated how often RC in pediatric IBD leads to actionable changes in treatment. Clarifying the impact of repeat endoscopic findings on treatment decisions may improve acceptance among both patients and providers.^8^ This study examines adherence to Nationwide Children's Hospital (NCH) institutional recommendations for RC within 15 months of diagnosis and the subsequent frequency and characteristics of treatment modifications following RC.

## 2 METHODS

### 2.1 Ethics statement

This study was approved by the NCH Institutional Review Board (STUDY00003173).

### 2.2 Study design

This single‐center retrospective cohort study conducted at NCH used an internal IBD patient database to identify patients (aged 0–21 years) with newly diagnosed IBD via colonoscopy between January 1, 2019 and December 31, 2021. Patients were excluded if they were not eligible for a RC at NCH within 1 year of diagnosis due to transfer of care or having undergone colectomy.

The study was developed in the context of ongoing local quality improvement (QI) work aimed at improving RC rates to assess for EH within 15 months of diagnosis. This practice is the recommendation and expectation among all providers at NCH, and performance on this metric is tracked over time through routine, transparent data reviews. Clinicians begin educating patients and caregivers at diagnosis that they will repeat the colonoscopy in approximately 1 year to assess for EH. This message is reiterated at yearly multidisciplinary IBD visits and supported by the use of a standardized, patient‐facing care pathway handout (Figure S1).

### 2.3 Data collection

Data were extracted from electronic medical records and included demographics, disease type (CD, UC, or IBD‐U), disease phenotype (via Paris Classification ^13^), treatment history, timing of RC, and therapy modifications after RC. All IBD treatments received by the patient were recorded from the time of their diagnostic colonoscopy to the 3 months after their RC (if completed). The date of RC (if completed) or reason for no RC (if identifiable via chart review) was recorded. The timing of RC and selection of IBD treatments were determined at the discretion of the patient's primary physician.

### 2.4 Outcomes

The primary outcome was the rate of RC within 15 months of diagnosis, aligning with the NCH institutional standard of treat‐to‐target monitoring RC by 15 months post‐IBD diagnosis. RC rate was grouped into three categories: early RC (≤6 months), standard RC (7–15 months), and delayed RC (>15 months).

The secondary outcome examined the frequency and types of therapy modifications after RC amongst those with an RC > 6 months from diagnosis. Since those undergoing early RC (≤6 months) did so in the context of suspected nonresponse to initial therapy rather than routine assessment of EH, they were excluded from these analyses. Therapy escalation after RC completion was defined as increasing the dose of a current medication, shortening the interval between doses of a current medication, switching to a new medication, including a biologic or small molecule, or adding a new medication (such as 5‐aminosalicylic acid [5‐ASA] agent or immunomodulator [IMM]) to the current regimen. Therapy de‐escalation was defined as decreasing the dose of the current medication, lengthening the interval between doses of current medication, or discontinuing IMM therapy (methotrexate or 6‐mercaptopurine/azathioprine). Each therapy modification category was mutually exclusive. Surgical intervention such as ileocecectomy after RC was a separate therapy modification; medication changes surrounding this surgery were excluded.

### 2.5 Statistical analysis

Quantitative data were summarized with medians and interquartile ranges (IQR, 25th–75th percentiles). Categorical variables were reported as frequencies (*n*) and corresponding percentages of the cohort. Group comparisons for categorical variables were conducted using the chi‐square test, with statistical significance defined as a two‐sided *p*‐value < 0.05. For comparative purposes, patients were divided into two groups: (i) those who had any therapy adjustment after RC and (ii) those without such changes. The chi‐square test was used to evaluate differences in demographic characteristics (e.g., race, sex) and clinical parameters (e.g., IBD subtype) between the two groups. A Mann–Whitney *U* test/Wilcoxon rank‐sum test evaluated the differences in continuous variables (e.g., median age) between the two groups. Due to the small number of patients with IBD‐U, those with UC and IBD‐U were combined into a single group for analysis. Descriptive and inferential analyses were performed using Stata 14.0 (Stata Corp).

## 3 RESULTS

### 3.1 Study population

**Figure 1:**
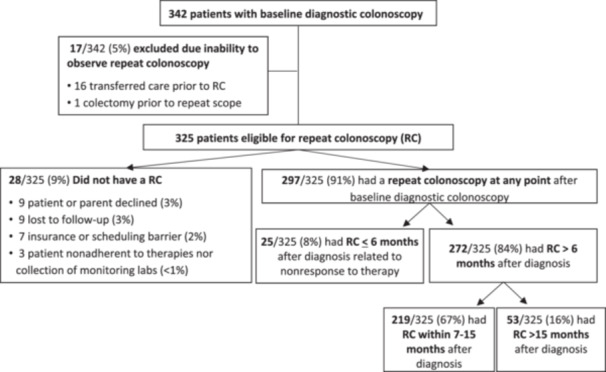
Patient flow chart. RC, repeat colonoscopy.

During the study period, 342 pediatric patients were diagnosed with IBD via a baseline colonoscopy at NCH. Seventeen patients (17/342; 5%) were excluded from analysis because they were not eligible for RC at NCH due to transfer of care to another institution (*n* = 16) or colectomy (*n* = 1) before 12 months after diagnosis (Figure 1). Thus, a total of 325 patients met the inclusion and exclusion criteria: 65% had CD (211/325) and 35% had UC/IBD‐U (114/325). The median age at IBD diagnosis was 14.5 years (IQR 11.6–16.9 years). Females comprised 46% (*n* = 148) of the included study patients. The racial distribution was 80% white, 11% Black or African American, 6% other or multiracial, 3% Asian or Pacific Islander.

### 3.2 Frequency of repeat colonoscopy

Overall, 91% of eligible patients (297/325) underwent RC at any time point post‐diagnosis. Among eligible patients, 8% (25/325) underwent RC within 6 months and were excluded from further analyses (Figure 1). Sixty‐seven percent of eligible patients (219/325) had a RC between the target range of 7–15 months, and 16% (53/325) had a RC beyond 15 months. Collectively, data analysis focused on the 272 patients who had an RC >6 months after diagnosis (Table 1). Within the delayed RC timeframe (>15 months), 83% of patients (44/53) had a RC between 16 and 23 months, with 17% (9/53) having a RC greater than 24 months after diagnosis (Table 2)**.** Nearly all patients (97%, 263/272) underwent their first RC within 2 years of diagnosis. Among those 272 patients with a RC at any interval >6 months after diagnosis, the median time to RC was 13.1 months (IQR: 11.5–15.4) (Table 2). Twenty‐eight patients did not have a RC at any time after diagnosis (28/325 [9%]).

**Table 1:** Characteristics of patients undergoing repeat colonoscopy in standard or delayed timeframe (>6 months after diagnosis).

	Total *n* = 272
Age at diagnosis (years)	
Median (IQR)	14.2 (11.3–16.4)
Sex (*n*, %)	
Female	126 (46)
Race (*n*, %)	
White	221 (81)
Black/African American	28 (10)
Other	15 (6)
Asian/Pacific Islander	8 (3)
Disease type (*n*, %)	
CD	183 (67)
UC/IBD‐U	89 (33)
Paris classification (*n*, %)	
CD	
Disease location	
L1	41 (22)
L2	29 (16)
L3	111 (61)
L4	2 (1)
Disease behavior	
B1	160 (87)
B2	5 (3)
B3	11 (6)
B2B3	7 (4)
Perianal disease	
Yes	28 (15)
UC/IBD‐U	
Extent of disease	
E1	14 (16)
E2	24 (27)
E3	6 (7)
E4	37 (42)
Undetermined	8 (8)
S1: Ever severe: Yes	23 (26)^a^

Abbreviations: B1, inflammatory; B2, structuring; B2B3, structuring and penetrating; B3, penetrating; CD, Crohn's disease; CD, Crohn's disease; E1, Proctitis; E2, Left‐sided UC (distal colon to splenic flexure); E3, extensive (distal colon to hepatic flexure); E4, pancolitis (proximal colon to hepatic flexure); IBD‐U, inflammatory bowel disease unspecified; IQR, interquartile range; IQR, interquartile range; L1, ileal only; L2, colonic; L3, ileocolonic; L4, isolated upper disease; UC, ulcerative colitis.

^a^ *N* = 81, undetermined IBD not included.

**Table 2:** Timing for RC among those with RC in standard or delayed timeframe (>6 months after diagnosis).

	Total *n* = 272
Time elapsed to repeat scope (months)	
Median (IQR)	13.1 (11.5–15.4)
Time period to repeat scope (*n*, %)	
7–15 months	219 (81%)
16–24 months	44 (16%)
>24 months	9 (3%)

Abbreviations: IQR, interquartile range; RC, repeat colonoscopy.

### 3.3 Failure to repeat colonoscopy

Reasons for no RC among these 28 patients included patient/caregiver refusal (*n* = 9; 32%), loss to follow‐up (*n* = 9; 32%), insurance or scheduling barrier (*n* = 7; 25%), and patient nonadherence to therapies and/or collection of monitoring labs leading the treating physician to delay RC (*n* = 3; 11%) (Figure 1).

### 3.4 Therapy modifications following repeat colonoscopy

Among the 272 patients with RC >6 months postdiagnosis, 165/272 (61%) had no subsequent changes in IBD management. In contrast, 107/272 (39%) underwent therapy modifications. Management changes after RC in these 107 patients included medication escalation (*n* = 87; 81%), medication de‐escalation (*n* = 17; 16%), and surgery (ileocecal resection) (*n* = 3; 3%) (Table 3). No surgical interventions occurred for patients with UC/IBD‐U.

**Table 3:** Therapy modifications following RC.

	Total (*n* = 272)	
	*n*	%
Patients with therapy changes after RC	107	100%
Types of therapy change after repeat scope	*n*	%
Escalation	87	81
Initiation of new biologic or small molecule	39	45
Decreased interval of current medication	21	24
Increased dose of current medication	12	14
Addition of medication to current regimen	12	14
Both of the above	3	3
De‐escalation	17	16
Increased interval of current medication	7	41
Discontinued immunomodulator	7	41
Decreased dose of current medication	2	12
Both increased interval and discontinued IMM	1	6
Ileocecectomy	3	3

*Note*: Each category is mutually exclusive. Abbreviations: IMM, immunomodulator; RC, repeat colonoscopy.

### 3.5 Therapy escalations

Of the 87 patients with a medication escalation post‐RC, treatment adjustments included initiating a new biologic or small molecule medication (39/87; 45%), adjusting the dose and/or interval of a current medication regimen (36/87; 41%), and adding another medication to their current regimen (12/87, 14%).

### 3.6 Therapy de‐escalations

Of the 17 patients with medication de‐escalation following RC, treatment adjustments included transitioning from combination antitumor necrosis factor (TNF) and IMM therapy to anti‐TNF monotherapy (7/17; 41%), lengthening the interval between anti‐TNF doses (7/17; 41%), or both discontinuing the IMM and increasing the interval between anti‐TNF doses (1/17; 6%).

### 3.7 Variables impacting therapy modifications

Demographic and clinical characteristics were compared between patients who underwent management changes and those who did not. No statistically significant differences were observed between groups in terms of median age at diagnosis, gender, race, and IBD type.

## 4 DISCUSSION

This single‐center study demonstrates a high rate of RC among children with IBD, with 91% undergoing RC at some point after diagnosis and two‐thirds of patients completing the repeat procedures within the intended interval of 7–15 months after diagnosis, consistent with the local standard of care. Given that clinical symptoms, laboratory and stool studies can be inadequate to assess disease burden fully, RC in this timeframe is recommended in the STRIDE II guidelines as part of a treat‐to‐target approach.^2^ Timing of RC within a defined follow‐up interval from diagnosis has not been a primary focus of prior pediatric IBD studies. To our knowledge, this is the first to report adherence rates in pediatrics following the widespread adoption of the STRIDE II treat‐to‐target recommendations.^2^ Other pediatric studies focused on EH rates and were limited to either those in clinical remission or those on anti‐TNF therapy.^14^, ^15^ Our study highlights the feasibility of implementing guideline‐based endoscopic monitoring amongst allcomers in a pediatric IBD population.

In contrast to commonly described barriers to RC in children, patient or caregiver refusal was rare, with only 3% declining RC for this reason, thus challenging assumptions about reluctance towards RC.^10^, ^11^ The low rate of patient refusal suggests that patient education and setting expectations for families at the time of diagnosis, along with standardized care pathways within a treat‐to‐target approach, may help overcome potential resistance to repeat colonoscopies and improve adherence to clinical guidelines.

While previous studies have demonstrated that RC can influence management decisions, they did not evaluate adherence to a standardized postdiagnosis interval or describe treatment changes in detail.^14^, ^16^ In our cohort, treatment changes following RC were common, with 39% of patients undergoing therapy adjustments, including both escalation and de‐escalation of therapy. Among those with a treatment change, 81% underwent medication escalation (started a new medication, increased the dose of current medication, shortened the current medication interval, or a combination of these adjustments). By comparison, Thakkar et al. reported treatment modifications in 42% of 230 patients undergoing RC and new medication initiation in 72% of these cases.^16^ Similarly, Santha et al. found that 28% of 104 pediatric IBD patients believed to be in clinical remission required escalation of therapy after RC.^14^ Our data confirm a similar frequency of therapy modifications after RC and expand upon these observations in a larger cohort. Specifically, our study adds greater detail on types of therapy modifications after RC, including dose intensification, interval shortening, and initiation of new medications.

Notably, 17% of those who underwent a therapy adjustment after RC had medication de‐escalation (decreased medication dose, increased medication interval, or transitioning from an anti‐TNF/IMM combination therapy to anti‐TNF monotherapy). Prior studies have not described de‐escalation in this context, highlighting the unique contribution of this study. These findings suggest that the assessment of EH via RC may support the safe reduction of medical therapies in select patients, minimizing medication‐related risks while preserving disease control. Data in both adult and pediatric settings suggest that medical therapy can be safely de‐escalated in specific scenarios.^17^, ^18^, ^19^, ^20^, ^21^, ^22^ Additional research is needed to define safe and effective strategies for dose reduction. RC plays a central role in driving personalized care by identifying patients who would benefit from either intensified therapy or de‐escalated medication regimens, as well as identifying patients with stricturing disease requiring surgical intervention, a finding that may not be definitively detected by biochemical testing or imaging.

While our study benefited from a large, well‐defined cohort with minimal attrition and detailed clinical data, we recognize that it has several limitations inherent to its retrospective design. While reporting rates of EH at the time of RC is an outcome of clinical interest, we were unable to report on this outcome due to incomplete use of and documentation of endoscopic scoring systems by providers, lack of video recording, and broad variation in reporting subtle endoscopic differences, thus limiting the ability for centralized review. Centralized review would have been limited to photos captured during the procedure that are of variable quality and insufficient to confidently describe rates of EH. While one could speculate that patients without medication changes and/or those with medication deescalation were found to have EH, this conclusion cannot be confidently assumed. Similarly, while we attribute therapy changes to the results of the RC, they could have occurred around the time of the RC due to medication intolerance or side effects, patient preference, insurance coverage, comorbid conditions, or a need for a modality change (from infusion to injection, for example). Our high percentage of patients completing RC after diagnosis likely reflects the NCH divisional quality improvement efforts including the development of tracking and reminder systems which emphasize the importance of RC to assess for EH within 15 months of diagnosis. Therefore, our single‐center findings may not be generalizable to broader pediatric IBD populations. However, we do feel that our results offer a compelling benchmark for what is achievable in pediatric IBD care.

Finally, we acknowledge that there are additional tools beyond colonoscopy to assist in the assessment of disease activity including fecal calprotectin, cross sectional imaging such as enterography, and intestinal ultrasound (IUS) each of which have inherent benefits and detractors. Fecal calprotectin is noninvasive, and levels less than 100–250 μg/g have been associated with endoscopic healing.^23^ Enterography also does not require anesthesia and has value in assessing small bowel inflammation as well as transmural healing. IUS is increasingly being used to noninvasively assess both mucosal and transmural inflammation, and while not a formal target in the STRIDE‐2 guidelines, likely will be in future iterations.^2^, ^24^ That said, colonoscopy remains the gold standard for assessment of endoscopic healing.

## 5 CONCLUSIONS

In conclusion, adherence to a treat‐to‐target strategy utilizing timely RC after IBD diagnosis was high, with rare patient/caregiver refusal. Treatment adjustments, including both therapy escalation and de‐escalation, were common, highlighting the value of endoscopic reassessment in guiding treatment optimization. Standardized care pathways can promote adherence and enhance clinical decision‐making.

## CONFLICT OF INTEREST STATEMENT

The authors declare no conflict of interest.

## Supporting information

Supplemental Figure 1. Example new diagnosis care pathway from Nationwide Children's Hospital.
