# Supplementary material for: Frequency and impact of repeat colonoscopy as a treat‐to‐target approach in pediatric inflammatory bowel disease
Source: JPGN Rep. 2025 Nov 11;7(1):70–6. doi: 10.1002/jpr3.70110 (PMC12894090; doi:10.1002/jpr3.70110)
Supplement: Supplementary file 1 — Supplemental Figure 1. Example new diagnosis care pathway from Nationwide Children's Hospital. [file JPR3-7-70-s001.docx]

Supplemental Figure 1. Example new diagnosis care pathway from Nationwide Children’s Hospital
